# Regulation of stem cell fate by HSPGs: implication in hair follicle cycling

**DOI:** 10.1038/s41536-022-00267-y

**Published:** 2022-12-28

**Authors:** Charlie Colin-Pierre, Oussama El Baraka, Louis Danoux, Vincent Bardey, Valérie André, Laurent Ramont, Stéphane Brézillon

**Affiliations:** 1 Université de Reims Champagne-Ardenne, SFR CAP-Santé (FED 4231), Laboratoire de Biochimie Médicale et Biologie Moléculaire, Reims, France; 2 CNRS UMR 7369, Matrice Extracellulaire et Dynamique Cellulaire-MEDyC, Reims, France; 3 BASF Beauty Care Solutions France SAS, Pulnoy, France; 4 CHU de Reims, Service Biochimie-Pharmacologie-Toxicologie, Reims, France

**Keywords:** Stem cells, Growth factor signalling

## Abstract

Heparan sulfate proteoglycans (HSPGs) are part of proteoglycan family. They are composed of heparan sulfate (HS)-type glycosaminoglycan (GAG) chains covalently linked to a core protein. By interacting with growth factors and/or receptors, they regulate numerous pathways including Wnt, hedgehog (Hh), bone morphogenic protein (BMP) and fibroblast growth factor (FGF) pathways. They act as inhibitor or activator of these pathways to modulate embryonic and adult stem cell fate during organ morphogenesis, regeneration and homeostasis. This review summarizes the knowledge on HSPG structure and classification and explores several signaling pathways regulated by HSPGs in stem cell fate. A specific focus on hair follicle stem cell fate and the possibility to target HSPGs in order to tackle hair loss are discussed in more dermatological and cosmeceutical perspectives.

## Introduction

Hair follicles (HFs) are mini-organs under the skin allowing to hair shaft growth^1^. A HF can be divided into three parts. The infundibulum is the upper portion between the skin surface and the sebaceous duct outlet. This duct connects the infundibulum of the hair follicle to the sebaceous gland, allowing the excretion of sebum along the hair shaft to hydrate the scalp. The middle part of the HF is the isthmus which extends from the sebaceous duct to the bulb. It is formed by different concentric layers forming the canal where the hair shaft grows^1^, from the outermost to the innermost layer: the connective tissue sheath, the outer root sheath (ORS), and the inner root sheath (IRS). This layer is in contact with the cuticle of the hair shaft until the level of the sebaceous canal where it disappears. The bulb, the deepest portion of the HF, is composed the hair matrix which surrounds the dermal papilla^2^.

Over the course of its life, hair follicle undergoes cyclic changes^3^. Forty to one hundred hairs are lost per day. Their renewal occurs during the hair growth cycle which is characterized by three main phases: anagen, catagen and telogen. An additional phase, called exogen phase, is controlled separately and leads to hair shaft loss^4,5^. The anagen phase allows the generation and growth of new hair shafts^6^. It lasts between three to 6 years on the average, divided into six stages. This phase is characterized by a remodeling of the HF morphology due to the activation (at the end of the telogen phase) and the intense proliferation of different cell types^7,8^. During the next catagen phase, the hair shaft stops growing and the transient segment of HF regresses^8^. It is divided into eight stages^8^ and lasts between 15 and 20 days. During this phase, apoptosis of keratinocytes is observed in a localized area, particularly at the junction of the secondary hair germ (SHG) and the dermal papilla (DP)^8^. The telogen phase is characterized by a dormant state of the DP and hair follicle stem cells making it a resting phase^8^. During this phase, the hair shaft remains anchored in the hair follicle^6^. After several months, the HF will return to the anagen phase thanks to stimuli.

The hair growth cycle is centered on the activation of HF pluripotent cells to differentiate and to provide the different cell lineages of the HF. Indeed, the hair shaft formation and the HF remodeling involve hair stem cells, located at the bulge, which contribute to generate the different cell lineages of the sebaceous gland, epidermis, and HF^9^. The process of the hair stem cell differentiation is complex and its study has revealed different populations derived from the hair stem cells. Five major distinct cell populations are defined in the literature based on their location, provenance, cell fate during the cycle phases, and their cellular markers: bulge stem cells, ORS progenitor cells, SHG progenitor cells, matrix transit-amplifying (TA) cells, and terminally differentiating cells (of the hair shaft, IRS and ORS)^10–13^. Moreover, the process of the hair stem cell differentiation involves many other cell types and specific niches: keratinocytes, fibroblasts of the DP, endothelial cells, fat cells, and immune system cells. The set of possible interactions between these different cell types complicates the study of the regulation of hair stem cell differentiation during the hair growth cycle.

All these cellular interactions are still poorly characterized, but it is known that growth factors (GFs) regulate the passage between the different phases of the hair cycle^14^. In particular, for the telogen to anagen transition and the hair shaft growth, several GFs are involved (such as Wnts, bone morphogenic proteins (BMPs), hedgehogs (Hhs) and fibroblast growth factors (FGFs)). A fine regulation of the GFs involved in the hair shaft growth is essential for the process of the hair cycle. The mechanisms involved in the regulation of these growth factors are still poorly understood, but several studies suggest that heparan sulfate proteoglycans (HSPG) are involved. These studies have shown an evolution of HSPG expression and distribution on the HF according to the phases of the hair cycle^15–18^. It has been demonstrated that the morphogenesis of a correct hair shaft requires a complex control of HSPGs production and sulfation^19^. Moreover, HSPGs are known to regulate many GFs involved in tissue or organ development and regeneration, such as those described for regulating the hair cycle^20–25^.

The purpose of this review is to make an update of the pivotal role of HSPGs in stem cell fate. Moreover, a specific focus on hair follicle stem cell differentiation during hair shaft growth is reported. Further, applications of these findings in the context of alopecia are also discussed.

### HSPG structure, synthesis, trafficking and location

#### Structure of glycosaminoglycans and proteoglycans

**Fig. 1:**
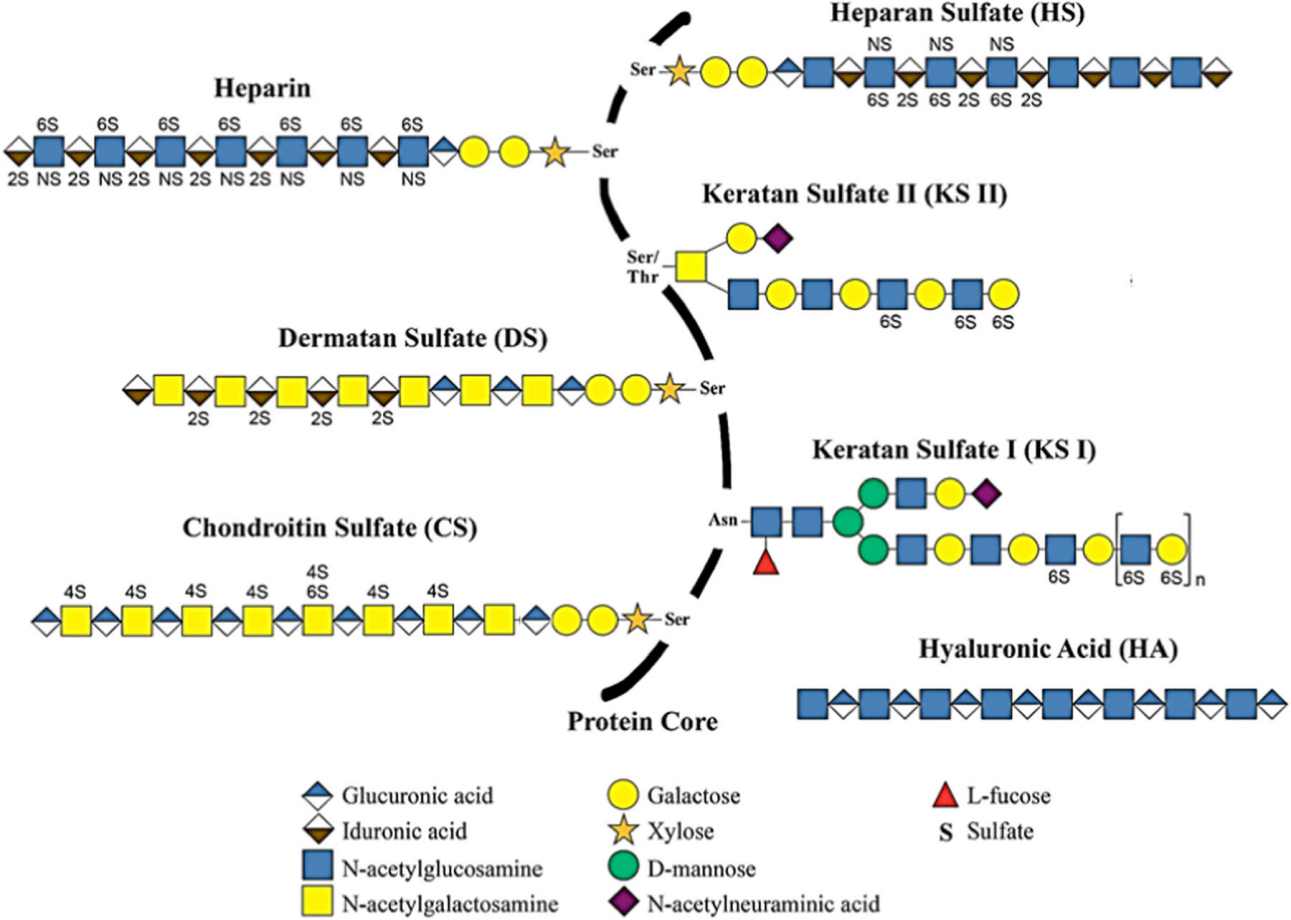
Representation of the structure of glycosaminoglycans, their sulfation sites and their covalent attachments to the core protein. The composition of the disaccharide unit repeats is schematically illustrated for heparan sulfate (HS), heparin, dermatan sulfate (DS), keratan sulfates I and II (KS I and II), chondroitin sulfate (CS) and hyaluronic acid (HA). Hyaluronic acid is the only GAG in free and unsulfated form. All the other GAGs are attached to a protein and present O-sulfation at the 2 (2 S) or 4 (4 S) and/or 6 (6 S) carbons, and/or N-sulfation (NS). (Figure adapted from Merida-de-Barros et al. 2018^29^; Copyright Elsevier).

In this section, the structure, the synthesis, the trafficking and the location are briefly described and summarized in Table 1. Proteoglycans (PGs), components of the extracellular matrix (ECM) and cell membranes, are produced by many cell types^26^. They are composed of a core protein to which one or more linear polysaccharide chains of glycosaminoglycans (GAGs) are covalently attached^27^. GAG chains are composed of a repeat of disaccharide units formed from a hexosamine (N-acetyl-glucosamine or N-acetyl-galactosamine) and an uronic acid (D-glucuronic acid or L-iduronic acid) or a single ose^27^. The detailed structure of the six different types of GAG has been already described^27–30^ and is illustrated in Fig. 1.

**Table 1:** Type, specific structural characteristics, and main roles of HSPG.

Location	Classification	Name	Specific structural characteristics	Roles	References
Cytoplasm	Secretory granules	Serglycin	Small core protein Serine-glycine repeats	Hematopoietic cell protease activity Inflammatory response	Avraham et al.^44^ Iozzo et Schaefer ^45^, Korpetinou et al.^46^, Kjellén et al.^47^, Schick et Senkowski-Richardson^48^, Kolset et al.^49^, Kolset et Tveit ^50^, Schick et al.^51^, Schick et al.^52^, Yurt et al.^53^, Pejler et al.^54^, Manou et al. 2020a, Bouris et al.^56^, Manou et al. 2020b
Membrane	Transmembrane	Betaglycan	Presents two forms: with or without GAG chains	Growth factor co-receptors	Couchman^58^, Karamanos et al.^59^.
		Neuropilin-1			
		CD44			
		Syndecans	Specific motif (cleavage zone) Variable region and two highly conserved domains	Cell processes (adhesion, cytoskeletal remodeling, migration) Growth factor co-receptors	Bernfield et al.^60^, Gondelaud et Ricard-Blum 2019, Kimet al. 1994, Choi et al.^63^, Chung et al.^64^, Echtermeyer et al.^65^, Leadbeater et al.^66^, Noguer et al.^67^
	GPI-anchored	Glypicans	Unique motif of 14 cysteine residues Highly conserved serine/glycine repeats	Regulation of cell signaling during tissue or organ development and regeneration	Filmus et al.^23^, Karamanos et al.^59^, Fransson ^68^, Veugelers et al.^69^, Hancock^71^, Mertens et al.^72^, Traister et al.^73^ Kawahara et al.^74^, Hereld et al.^75^, McGough et al.^76^
Pericellular	Basement membrane	Perlecan	Laminin-like domain	Integrity and function of basement membranes	Iozzo et Schaefer ^45^, Costell et al.^77^, Iozzo^78^, Amenta et al.^79^, McCarthy^80^, French et al.^81^, Mazzon et al.^82^, Mazzon et al.^83^.
		Agrin			
		Type XVIII collagen	Thrombospondin-like domain		
		Type XV collagen			
Extracellular	SPOCK	Testicans	Composed of five domains	Regulation of the CNS development Adipose tissue maturation	Iozzo et Schaefer ^45^, Vannahme et al.^84^, Kohfeldt et al.^85^, Bonnet et al.^86^, Hartmann et al.^87^, Charbonnier et al.^88^, Schnepp et al.^89^, Yamamoto et al.^90^, Alshargabi et al.^91^.

*CNS* central nervous system; *GAG* glycosaminoglycan.

Due to their structure and composition, GAGs are capable of binding to many components^31–37^.

The nature of the HS GAG chains, in addition to the amino-acid sequence of the core protein, defines the characteristics and properties of the HSPGs as well as their trafficking.

#### Biosynthesis and trafficking of HSPGs

HS biosynthesis takes place in the Golgi apparatus^38^. The HS GAG chain is formed in a stepwise manner by glycosyltransferases and sulfotransferases^39^. They use as substrate uridine diphosphate (UDP)-sugar and 3'-phosphoadenosine-5'-phosphosulfate (PAPS), respectively.

The first step of GAG chain initiation on core protein is the formation of the “linker”^38^. This step consists of the binding of a xylose to a serine of the core protein by xylose transferase. Then the addition of the other three monosaccharides forming the tetrasaccharide unit is catalyzed by galactosyl transferase I and glucuronic acid transferase I^39^. The next step consists of the elongation of the HS GAG chain by the addition of repeated disaccharide units^38,39^. Studies have shown that several factors influence this step and predetermine the type of GAG produced. For example, the amino-acid sequence surrounding the serine residue where the GAG chain covalent binding occurs;^40,41^ the production and transport of UDP-sugars in the Golgi apparatus;^39^ or the “linker” phosphorylation, epimerization or sulfation^42,43^.

Once the HSPGs have been synthesized, they are transported to the membrane or secreted to the ECM (except serglycin which is cytoplasmic). These two processes are influenced by different conditions (pH, specific amino-acid sequence, nature of sugars, glycosylphosphatidylinositol (GPI) anchor…). Studies, conducted on polarized cells, show that HSPGs are sorted to be secreted basolaterally to bind to the plasma membrane or apically to join the ECM^39^.

#### Classification of HSPGs according to their location

There are three main families of proteoglycans differing in their location. HSPGs are either cytoplasmic, either bound into the cell membrane, or composing the basement membrane or secreted in the ECM.

#### Cytoplasmic HSPGs

Only one PG belongs to the cytoplasmic PG family: serglycin. It is composed of a small core protein, about 16 kDa^44^, characterized by serine-glycine repeats^45^ and different GAG chains depending on the cell type^46^. Most commonly, serglycin has GAG chains of HEP and/or CS^47^ but can also rarely exhibit GAG chains of HS and CS^48^.

Serglycin is found in different cell types of hematopoietic origin such as cytotoxic T lymphocytes, neutrophils, and eosinophilic polymorphs^49^. It is also detected in macrophages^50^, endothelial cells^51^ and in embryonic stem cells^52^. When associated with HEP or HS chains, this PG is predominantly present in mast cells rich in secretory granules^50,53^.

Serglycin plays an important role in hematopoietic cell protease activity^54^. Indeed, it regulates the storage of proteases in the secretory granules as well as their protease activity during their intracellular release. It also plays a role in the immune response, in particular in the inflammatory response, by interacting with many components of the immune system^54^. Moreover several studies have shown the pro-tumorigenic functions of the serglycin^55^ and its ability to induce epithelial to mesenchymal transition^56,57^.

#### Membranes HSPGs

Membrane HSPGs are composed of several members grouped into two major families: transmembrane and GPI-anchored HSPGs^45^.

**Fig. 2:**
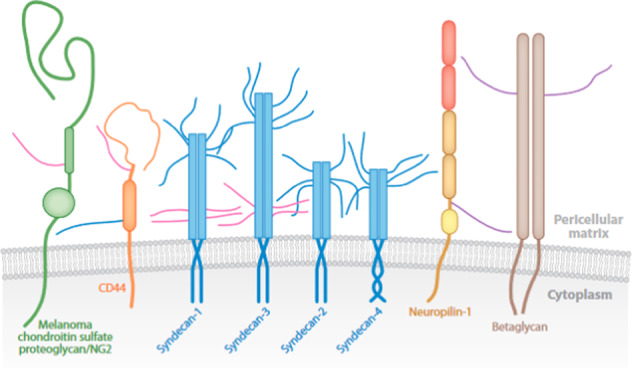
Schematic representation of transmembrane proteoglycans. The different proteoglycans of this family are represented with their GAG chains (CS in pink, HS in blue, HS or DS or CS in purple). (Figure from Couchman, 2010^58^; Copyright Anal Review license).

The transmembrane HSPG family is largely represented by syndecans (SDCs), and three other members: betaglycan, neuropilin-1 and CD44^58,59^ (Fig. 2). The three latter’s can occur in two forms: with or without GAG chains that may be HS and/or CS or DS^58^. They regulate many cell signaling pathways as GF co-receptors^58,59^.

The SDC group is composed of four members: SDC1 to 4, in order of discovery. They differ from each other largely by their extracellular domain, which is the most variable area of the core protein, with only 10-20% similarity^60^. Within the highly conserved amino-acid sequence of SDC family, there is a specific motif, close to the transmembrane domain, that is recognized by proteases responsible for cleaving the extracellular domain of SDCs and sequences involved in the attachment of GAG chains^61^. All four syndecans have HS chains at their N-terminus. In addition, SDC1 and 3 also carry CS chains (Fig. 2). The transmembrane and cytoplasmic regions of the syndecans are highly homologous, with 60 to 70% sequence homology^60^. The cytoplasmic domain is characterized by a variable region V surrounded by two highly conserved domains C1 and C2. These two domains are involved in functions common to all the four SDCs, whereas the V domain has a role in the specific functions of each syndecan.

SDCs are present on the surface of most of the cells and their expression is finely regulated. Depending on the stage of cell, tissue or organ differentiation, the four syndecans are expressed differently and selectively, reflecting their distinct function^62^. SDCs regulate many cell processes such as adhesion, cytoskeletal remodeling, and migration^63^. They act as co-receptors for several cell signaling pathways such as FGF2, vascular endothelial growth factor (VEGF), granulocyte-macrophage colony-stimulating factor (GM-CSF) or hepatocyte growth factor (HGF)^63^. They are also known for their implication in tissue repair and regeneration such as wound healing, vascular or neuronal repair^64–67^.

**Fig. 3:**
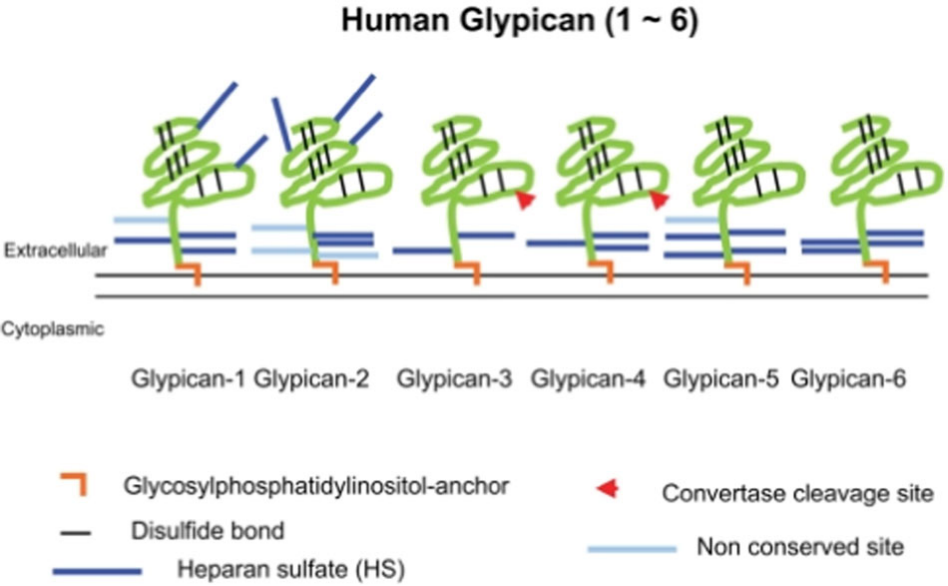
Schematic representation of the six glypicans. The core protein (green) is connected to the membrane by a GPI-anchor (brown) and the GAG chains (light and dark blue) on the core proteins are represented. Arrowheads represent the cleavage site of the core protein by convertase. (Figure from Yoneda et al., 2012^252^; Copyright Journal of Histochemistry and Cytochemistry license).

Glypicans (GPCs) form the GPI-anchored proteoglycan family^68^. It is characterized by a unique motif of 14 cysteine residues, which is conserved in all GPCs, including those of Drosophila, Dally and Dally-like-protein (Dlp)^23^. In human, six GPCs have been identified, GPC1 to 6 (Fig. 3). They are divided into two subfamilies: GPC1, 2, 4 and 6 (related to Dlp) and GPC3 and 5 (related to Dally). These two subgroups present 25 % of similarity including the sequences of the insertion site of HS type GAG chains^23^. These sites are composed of highly conserved serine/glycine repeats located near the C-terminus^23,69^. The GAGs of the glypicans are exclusively HS type except for GPC5 which can also have CS-type GAG chains. All GPCs have a core protein of ~60 kDa. They are expressed by many tissues and cells. Usually in polarized cells, GPI-anchored proteins are found in lipid rafts located at the apical pole^70^, involved in various cell signaling^71^. In these cells, GPI-anchored GPCs can also be present in large quantities at the basolateral region^72^. Interestingly, it has been shown that unglycosylated GPCs are localized at the apical pole, demonstrating that HS chains play a role in the trafficking of GPCs^72^. GPCs can be cleaved by different molecules such as Notum^73^, disintegrin and metalloproteinase 17 (ADAM17)^74^ or phospholipase C (PLC)^75^. GPCs play a role in modulating cell signaling during tissue or organ development and regeneration by regulating many cell signaling pathways such as Wnt, Hh, BMP, and FGF^23,59,76^.

#### Pericellular HSPGs

There are four pericellular PGs or basal membrane PGs: perlecan, agrin, type XVIII and type XV collagens. These PGs exhibit HS chains but, the type XV collagen can also have CS chains. They are associated with laminin or type IV collagen allowing the integrity of basement membranes^77,78^. They can also be associated with the cell surface *via* integrins^45^ ensuring signal transmission^79^, an essential function of the basement membrane. Moreover they are essential for embryogenesis and tissue maturation^80^. For example, perlecan promotes the chondrocyte differentiation^81^ and agrin plays a crucial role in the hematopoietic niches^82^ and monocyte maturation^83^.

#### ECM HSPGs

The ECM HSPGs are represented by the testican family. They are also named secreted protein acidic and cysteine rich (SPARC)/Osteonectin and Kazal-like domain proteoglycans (SPOCKs). This family is composed of three members: testican 1, 2 and 3. Their other name, SPOCK, is due to their characteristic protein domains. Their core protein is composed of five domains^84^. The N-terminal domain I is specific to the testican family, hence the name testican-specific domain, and corresponds to a signal peptide^84^. The domain II is called follistatin domain and is a module rich in cysteine. The domain III is characterized by binding sites of low affinity to calcium^85^, earning it the name of calcium binding domain. The domain IV, or thyroglobulin domain, contains a tetrapeptide sequence CWCV stabilized by three disulfide bridges corresponding to a thyroglobulin-like domain^45^. The domain V, also specific to this family, contains the two potential binding sites for GAG chains.

The GAG chains of SPOCKs are predominantly of the HS type^45^. They are expressed almost exclusively in the brain^84,86,87^ and studies suggest that they play a regulatory role during central nervous system development^88–90^. A recent study has shown that SPOCK1 induces adipocyte differentiation and adipose tissue maturation through the up-regulation of adipogenesis-related genes^91^.

#### Role of HSPGs in the regulation of stem cell differentiation during morphogenesis and regeneration of organs

**Fig. 4:**
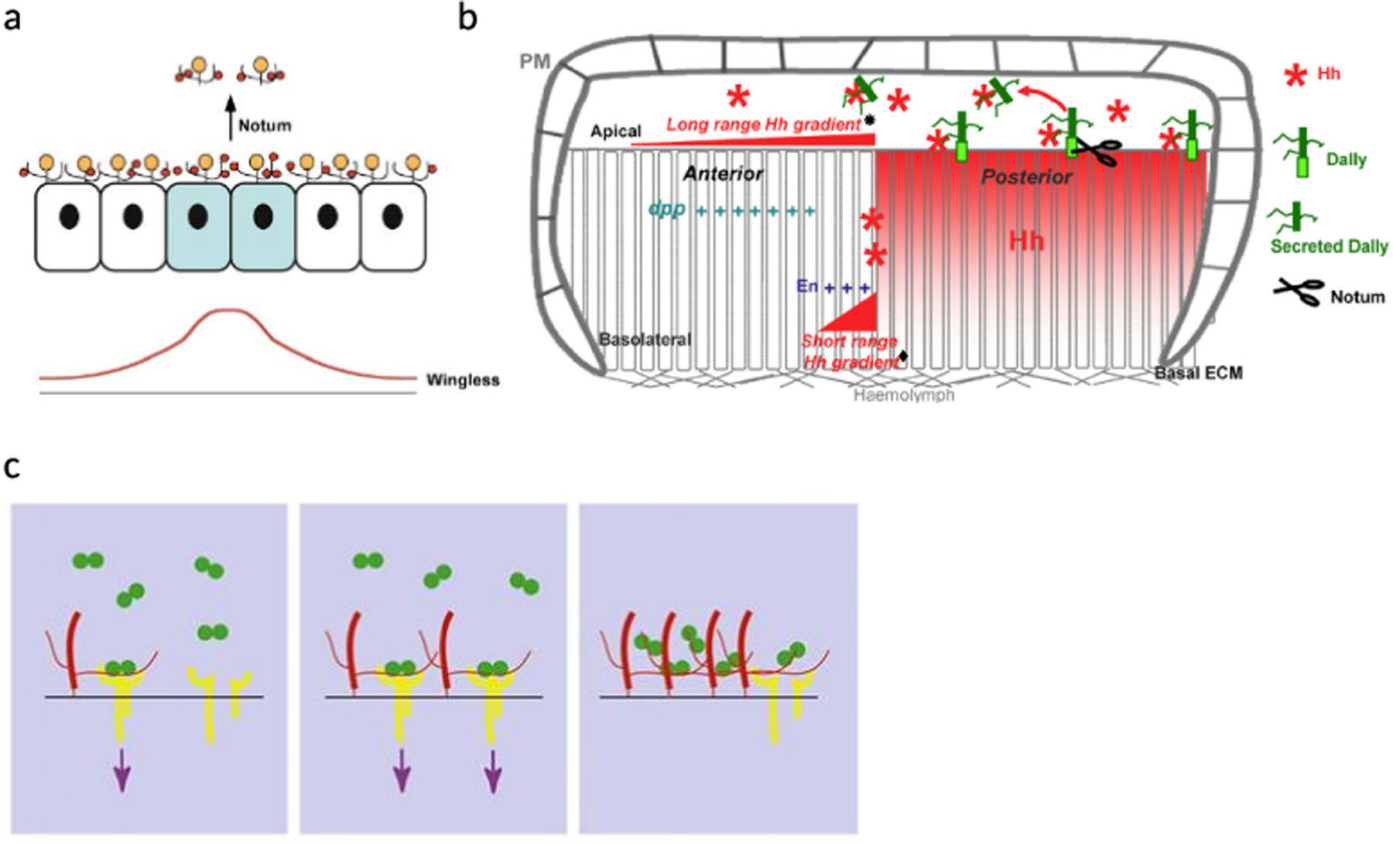
Drosophila glypicans regulate Wnt, Hh and BMP signaling pathways during Drosophila embryonic development. **a** Dlp regulates the activation of the Wnt pathway and the establishment of the dorsoventral (DV) axis during Drosophila embryonic development. Wg growth factor (red) and Notum enzyme are produced by the cells at the DV boundary (marked in blue). Dlp (yellow) sequestrates Wg and Notum cleaves the GPI anchor of Dlp. These processes lead to the formation of the Wg gradient (red line). (Figure from Kreuger et al., 2004^24^; Copyright Elsevier license). **b** Dally (green) regulates Hh (red star) activity during the establishment of the anterior-posterior axis during Drosophila embryonic development. Hh is secreted by future posterior cells and a short-range gradient (♦) is formed by diffusion of Hh to the future anterior cells (basolateral level). A long-range Hh gradient (∗) is enabled by Hh-related Dally cleavage by Notum at the apical pole of the cells. ECM extracellular matrix; PM plasma membrane. (Figure from Ayers et al., 2010^20^; Copyright Elsevier license). **c** Regulation of the activation of Dpp signaling pathways by Dally. Left: Dally (red) favors the interaction between Dpp (green) and its receptor (yellow) allowing the activation of the signaling pathway (purple arrows). Middle: This activation can be enhanced by an augmentation of Dally levels. Right: An excess of Dally levels leads to an inhibition of the signaling pathway activation via the sequestration of Dpp. (Figure reproduced with permission of the journal Development from Fujise et al., 2003^151^).

In this section, the role of HSPGs in the regulation of major signaling pathways known to be involved in embryonic and adult stem cell differentiation are presented (Table 2). The stem cell differentiation needs to be finely regulated during the development or the regeneration of organs. Several studies have shown the role of HSPGs in this orchestrated process. The mechanism of action of HSPGs is well described in numerous studies conducted on Drosophila. Indeed, it is a powerful model to understand the complex processes involved in human stem cell fate because they present very high homology with human HSPGs (Fig. 4).

**Table 2:** Role of HSPGs in the regulation of major signaling pathways involved in embryonic and adult stem cell fate.

Cells	HSPGs	Growth factors/Signaling pathways	Promoting effect of HSPGs on stem cell fate and organ formation/regeneration/homeostasis	References
Drosophila embryonic stem cells	Dlp	Wg	Establishment of the dorso-ventral axis	Kreuger et al.^24^
	Dally	Shh	Establishment of the anterior-posterior axis	Ayers et al.^20^
		Dpp	Development of wing	Akiyama et al.^149^, Fujise et al.^151^
	Trol	Wg	Formation of pre- and postsynaptic structures	Kamimura et al.^106^
		Wg and Dpp	Formation of second instar brain	Lindner et al.^107^
		Hh	Differentiation of neural stem cell	
		Hh and FGF	Activation of neural stem cell division	Park et al.^134^
Vertebrate embryonic stem cells	HS		Maintenance of embryonic stem cell pluripotency (attachment and growth)	Stelling et al.^188^
		BMP and FGF	Differentiation of embryonic stem cell during mesoderm formation	Kraushaar et al.^152^
	HS sulfation	Wnt, BMP and FGF	Maintenance and differentiation of mouse embryonic stem cells	Sasaki et al.^110^
		FGF	Differentiation of embryonic stem cells into neural progenitor cells	Johnson et al.^168^
			Differentiation of embryonic stem cells	Hirano et al.^189^, Hirano et al.^190^
	Glypican-4	Wnt	Inhibition of embryonic stem cell differentiation	Fico et al.^108^
		Wtn and FGF	Migration of lateral line collective cell during zebrafish embryogenesis	Venero Galanternik et al.^109^
	Agrin	FGF	Formation of zebrafish retina	Liu et al.^169^
Neuroepithelial cells	HSPG		Essential before and during neurogenesis	Yamaguchi et al. 2001
		FGF	Differentiation of murine neuroepithelial tissue	Nurcombe et al.^173^, Brickman et al.^170^
	HS	FGF	Proliferation, survival and differentiation of neuroepithelial cells	Murphy et al.^172^, Guilemot and Zimmer 2011
	HS sulfation	FGF	Proliferation and differentiation of neural stem cell	Yamada et al.^174^
	Syndecan-1	Wnt	Maintenance and proliferation of neural progenitor cells during cortical neurogenesis	Wang et al.^111^
	Syndecan-3	HB-GAM	Facilitation of neuroblast migration during brain development	Raulo et al.^191^
		GDNF		Bespalov et al.^192^
	Syndecan-4		Regulation of neural stem cell proliferation during zebrafish neurogenesis	Luo et al. 2016
	Glypican-1	FGF	Control of the brain size during neurogenesis	Jen et al.^175^
	Glypican-4	FGF	Maintenance of murine neuroepithelial cells	Hagihara et al.^176^
		FGF	Regulation of forebrain patterning of Xenopus	Galli 2003
	Glypican-6	FGF	Development of mouse cerebral cortex	Salehi^178^
	Perlecan	FGF	Proliferation and differentiation of neural stem cells during neural tube formation	Joseph et al.^180^, Haubst et al.^179^, Giros et al. 2007
		Hh	Regulation of neurogenesis	Giros et al. 2007, Palma et al.^136^
	Agrin	Wnt	Differentiation of neuroepithelial cell during the formation of neuromuscular junctions	Henríquez and Salinas 2011
		Hh and FGF	Development of GABAergic and glutamatergic neuron in zebrafish brain	Zhang et al.^137^
Drosophila adult stem cells	HS sulfation	Hh, EGFR and Jak/Stat	Division and differentiation of intestinal stem cell during regeneration	Takemura and Nakato^138^
	Dlp and Dally	Wg, Hh and Jak/Stat	Maintenance of ovarian adult stem cells	Su et al.^113^
	Dally	Dpp	Regulation of stem cell number in germline stem cell niche	Hayashi et al.^153^, Dejima et al^150^.
Hematopoietic progenitor cells	HSPG/HS		Adhesion of stem and progenitor cells to stromal cells	Siczkowski et al.^193^, Zweegman et al.^194^
	HSPG	SDF-1	Migration, homing and retention of progenitor cells	Netelenbos et al.^195^
	HS	SDF-1	Migration of progenitor cells	Netelenbos et al.^198^
		GM-CSF	Promotion of haematopoiesis	Gordon et al.^196^
		Il-3	Regulation of hematopoietic lineage formation	Roberts et al.^197^
	HS/heparin	Il-6, PF4 and TGFβ	Differentiation of megakaryocyte progenitors	Han et al.^199^
Osteogenic and Chondrogenic progenitors	HS	BMP	Potentiation of bone repair	Bramono et al.^154^
		TGFβ	Promotion of chondrogenic differentiation	Chen et al^202^.
	HS/Heparin		Promotion of chondrogenesis and cartilage nodule formation	San Antonio et al.^200^
	Heparin	Wnt	Differentiation of osteogenic progenitor cell	Ling et al.^114^
	Syndecan-3	Hh	Proliferation and maturation of chondrocyte	Shimo et al.^142^
		BMP	Inhibition of chondrogenesis during cartilage differentiation	Fisher et al.^155^
		FGF	Proliferation of chondrogenic progenitors	Kirsch et al.^181^, Shimazu et al.^182^
	Glypicans-1 and -3	BMP	Inhibition of osteogenesis during bone regeneration	Dwivedi et al.^222^
	Glypican-3 sulfation		Regulation of osteogenic lineage formation	Haupt et al. 2009, Zhao et al. 2015
	Glypican-6	Hh	Growth of developing long bones	Capurro et al.^21^
	Perlecan		Promotion of chondrogenesis	Gomes et al.^201^
		BMP	Improvement of osteogenesis	Decarlo et al.^157^
			Stimulation of chondrogenic differentiation	Jha et al.^156^
	Agrin	BMP	Differentiation of osteoblast	Souza et al.^158^
		Wnt	Differentiation of chondrogenic stem cell during osteochondral regeneration	Eldridge et al.^115^
Intestinal progenitor cells	HS	Wnt	Differentiation of intestinal progenitor cells during the regeneration of intestinal crypt	Yamamoto et al.^25^
	HS sulfation		Regulation of colonic epithelial cell differentiation	Jao et al.^203^
Muscle satellite cells and myoblasts	HS	FGF	Regulation of muscle satellite cells and differentiation of myoblasts	Rapraeger et al.^184^, Olwin and Rapraeger^183^
	Syndecan-3	FGF	Inhibition of myogenic differentiation	Fuentealba et al.^185^
		Notch	Regulation of adult myogenesis	Pisconti et al.^204^
	Syndecan-4		Expression by self-renewing muscle stem cell	Tanaka et al. 2009
	Glypican-1	FGF	Formation of muscle	Gutierrez and Brandan 2010
Skin progenitor cells	HSPG		Evolution of its expression within the epidermis	Caughmman et al. 1987, Horiguchi et al.^206^
	Syndecan	TRPC	Regulation of adhesion, adherens junction composition, and early differentiation	Gopal et al.^208^
	Syndecan-1		Evolution of its expression within the epidermis	Sanderson et al.^207^
	Glypican-1		Evolution of its expression within the epidermis	Perrot et al.^187^
		FGF	Proliferation of keratinocyte progenitors	
Hair follicle stem cells and progenitors	HSPG		Expression structure dependent in the hair follicle	Bernard^218^, Botchkarev and Kishimoto^219^, Couchman^220^, Westgate et al.^221^
	HSPG sulfation		Necessity for correct morphogenesis of hair shaft	Coulson-Thomas et al.^19^
	Syndecan-1		Diminution of its expression in ORS during telogen phase	Bayer-Garner et al.^15^
	Glypican-1		Maintenance of its expression in the hair matrix and hair shaft along the hair cycle	Colin-Pierre et al.^215^
	Glypican-1 sulfation		Variation of the type and/or the degree of sulfation during hair cycle	
	Perlecan		Diminution of its expression in dermal papilla in late catagen phase	Malgouries et al.^17^
	Versican*	Wnt	Induction of anagen phase and hair inductivity	Yang et al.^224^
	Decorin*	TGFβ	Induction of anagen phase	Inui and Itami^225^

*HSPG* heparan sulfate proteoglycan; *Dlp* Dally-like-protein; *Wg* Wingless; *Hh* Hedgehog; *Dpp* BMP2 and 4 Drosophila homologous; *FGF* fibroblast growth factor; *HS* heparan sulfate; *BMP* bone morphogenic protein; *HB-GAM* pleiotrophin; *GDNF* glial cell line-derived neurotrophic factor; *EGFR* epithelial growth factor receptor; *SDF-1* stromal cell-derived factor-1; *GM-CSF* granulocyte-macrophage colony-stimulating factor; *IL* interleukin; *PF4* platelet factor 4; *TGF* transforming growth factor beta; *TRPC* transient receptor potential canonical channel; * other types of proteoglycan.

#### Implication of HSPGs in Wnt signaling pathways

The human Wnt family is composed of 19 members that bind to Frizzled transmembrane receptors (7 members) involving a coreceptor recruitment^92,93^. The Wnt signaling pathways were demonstrated to regulate the stem cell differentiation process occurring during embryogenesis and development/regeneration of numerous organs^94–105^. Wnts are able to induce different signaling pathways^93^ and to exhibit different effects on the stem cell fate, depending on the coreceptor with which Frizzled receptors are associated.

The regulation of Wnt signaling pathways is crucial for a correct differentiation of stem cell and thus for a correct morphogenesis or regeneration of organs. Some studies have shown the regulation of Wnt signaling pathways by HSPGs during the stem cell differentiation.

#### Regulation of Wnt signaling by HSPGs during Drosophila embryogenesis

The two first examples highlight the role of HSPGs during Drosophila embryogenesis. Kreuger and collaborators have studied the regulation of dorso-ventral axis establishment during Drosophila embryogenesis^24^. They were able to show that the glypican Dlp is involved in the formation of the Wingless (Wg) gradient necessary for the establishment of the dorso-ventral axis (Fig. 4a). Drosophila perlecan homologue Trol was shown to have an important role during Drosophila nervous system development^106,107^. Indeed, by regulating Wg signaling, it was demonstrated to regulate the formation of pre- and postsynaptic structures^106^ and a second brain instar^107^.

#### Regulation of Wnt signaling by HSPGs modulates vertebrate embryonic stem cell fate

In the case of vertebrate development, several studies have demonstrated the roles of HSPGs in the regulation of embryonic stem cells and neuroepithelial cells by modulating Wnt signaling. In particular, GPC4 regulates Wnt/β-catenin signaling inhibiting the differentiation of mouse embryonic stem cells and promoting their self-renewal^108^. GPC4 was also shown to play a role during zebrafish development^109^. Indeed, it regulates Wnt/β-catenin signaling to promote the essential migration of lateral line collective cells during embryogenesis. Moreover, Sasaki and collaborators have demonstrated the crucial role of HS sulfation for the regulation of self-renewal and differentiation of mouse embryonic stem cells^110^. A study conducted in vivo on mouse cortical neurogenesis has demonstrated that SDC1 promotes the activation of Wnt signaling pathways during neural progenitor cell differentiation^111^. This activation permits to maintain their phenotype and their proliferation potent. As last example, it has been shown that a collaboration between Wnts and agrin promotes cell differentiation for the formation of vertebrate post-synaptic neuromuscular junctions^112^.

#### Regulation of Wnt signaling by HSPGs modulates adult stem cell fate

Some studies have been conducted on adult stem cell differentiation to understand how HSPGs regulate the Wnt signaling pathways during regeneration of organs. In the case of Drosophila adult stem cells, Dally and Dlp are expressed in the niche of ovarian adult stem cells and play a role in the self-renewal of these cells by regulating different pathways including Wg^113^. As first example in the case of vertebrate adult stem cells, it has been shown that HSPGs promote the activation of Wnt signaling pathways by modulating the binding of Wnts on mouse intestinal progenitor cells^25^. The activation of the leucine-rich repeat-containing G-protein coupled receptor 5 (LGR5) progenitor cells induces their differentiation and leads to the regeneration of intestinal crypt. Similarly, in the context of osteoblast differentiation, the association of heparin with Wnt3a leads to murine osteogenic progenitor cell differentiation^114^. As third example, a study conducted on osteochondral regeneration has demonstrated the important role of agrin in the mouse chondrogenic stem cells by downregulation of Wnt/β-catenin signaling pathway^115^.

#### Implication of HSPGs in Hh signaling pathways

The human Hh family is composed of 3 members: Desert hedgehog (Dhh), Indian hedgehog (Ihh) and Sonic hedgehog (Shh). They are secreted and bind to Patched membrane receptor^116^. Several studies have demonstrated the involvement of Hhs in the regulation of vertebrate embryogenesis and vertebrate organs development/regeneration^117–121^. These studies have shown the role of Hhs in the regulation of stem cell differentiation. Moreover, it is known that Hh proteins can act at short or long-range, especially during embryogenesis, to regulate organ development (imaginal discs of Drosophila or vertebrate neural tubes for example)^122–126^.

Thus, the Hh short or long-range gradients need a fine regulation to be set up and allow a correct stem cell differentiation. Several studies have demonstrated the capability of HSPG to regulate the gradient and the effect of Hhs during morphogenesis or regeneration of organs.

#### Regulation of Hh signaling by HSPGs during Drosophila and vertebrate embryogenesis

In the case of embryogenesis, the establishment of Drosophila^127,128^, mouse^129,130^ or zebrafish^131^ mutant embryos deficient for UDP-glucose (required for GAG synthesis) or loss-of-function mutation for an HSPG provides evidence on the essential role of HSPGs in the regulation of Hh signalization during organ development. Moreover, some studies have demonstrated that the establishment of Hh gradients requires HSPGs^132,133^. For example, Ayers and collaborators have been able to demonstrate the involvement of the glypican Dally in the establishment of the Drosophila anterior-posterior axis thanks to the regulation of the Hh gradient (Fig. 4b). Future cells of the posterior zone secrete Hhs, two gradients are then formed: a short-range gradient at the basolateral level of the cells by limited diffusion and a long-range gradient at the apical pole due to the sequestration of Hhs by Dally and its release by Notum, allowing to transport Hhs to act over a long distance^20^. Drosophila perlecan homologue Trol was shown to induce the activation of neural stem cell^134^ and their differentiation into a specific neuroblast population by regulating Hh pathway^107^.

Some studies have demonstrated the implication of Hh signaling modulation by HSPGs during vertebrate development in particular in the case of brain formation. For example, perlecan has been shown to regulate mouse neurogenesis by mediation of Shh concentration gradient^135,136^ and agrin has been demonstrated to control neuron development in zebrafish brain by regulating Shh^137^.

#### Regulation of Hh signaling by HSPGs modulates adult stem cell fate

Several studies have revealed the importance of HSPGs on adult stem cell differentiation regulated by Hh signaling. In the case of Drosophila adult stem cells, Dally and Dlp regulate ovarian stem cell maintenance by regulating Hh signaling^113^ and the HS sulfation is essential for intestinal stem cell division and differentiation during regeneration^138^. Other studies conducted on the differentiation of osteogenic and chondrogenic progenitors in vertebrate have shown that the HS GAG chains are involved in the Hh long-range gradient formation^139–141^ essential for the regulation of chondrocytes differentiation. More precisely, Capurro and collaborators have demonstrated in vitro the capability of GPC6 to form a ternary complex with Hh and its receptor^21^. Indeed, GPC6 binds the Hh ligand with its core protein and the Hh receptor with its HS chains to promote their interaction and the long bone growth. SDC3 was also shown to be involved in chick chondrocyte proliferation and maturation by regulating Ihh signaling^142^.

#### Implication of HSPGs in BMP signaling pathways

The 20 secreted BMPs composed the human BMP family. They bind to transmembrane BMP receptors (BMPR)^143,144^. The signaling pathways of BMPs are involved in the regulation of vertebrate embryogenesis and vertebrate organs development/regeneration^145,146^ and they are particularly known to regulate bone formation and osteoblast differentiation^147,148^.

The regulation of the activation of BMP signaling pathways is crucial for a correct differentiation of stem cell and thus for a correct morphogenesis or regeneration of organs.

#### Regulation of BMP signaling by HSPGs during Drosophila and vertebrate embryogenesis

Several studies conducted on Drosophila embryos have shown the regulation of these pathways through the interaction between HSPGs and BMPs^149–151^. More precisely, as shown in Fig. 4c, Dally could regulate Dpp (BMP2 and 4 Drosophila homologous) through the stabilization of the interaction between Dpp and its receptor or the sequestration of Dpp^151^. Moreover, Dally could participate to the internalization and the degradation of the Dpp-receptor complex^149^ during wind development. Trol is known to play a role during Drosophila second brain instar formation by regulating Dpp in a Trol dependent manner^107^.

In the case of vertebrate embryogenesis, few evidence show the implication of HSPGs in the regulation of BMP signaling. In particular, the HS chain and its sulfation are crucial for a correct regulation of mouse embryonic stem cell differentiation^110^ and for mesoderm formation from mouse embryonic stem cells^152^ induced by BMP.

#### Regulation of BMP signaling by HSPGs modulates adult stem cell fate

Some studies have been conducted on adult stem cell differentiation to understand how HSPGs regulate the BMP signaling pathways in the context of organ homeostasis or regeneration. As first example, two studies conducted on Drosophila adult stem cells have demonstrated that Dally is the co-receptor of Dpp in the germline stem cell niche and it regulates the number of these stem cells^150,153^.

In the case of vertebrate adult stem cells, several studies performed on osteogenic and chondrogenic progenitors have revealed the role of HSPGs in the regulation of BMP signaling involved in cell maintenance or differentiation. HS chains have been shown in vitro to potentiate the BMP2-induced bone repair by prolonging BMP-2 half-life, reducing interactions between BMP-2 with its antagonist noggin, and modulating BMP2 distribution on the cell surface^154^. SDC3 has been demonstrated in vitro to impair the interaction between BMP2 and its receptors leading to an inhibition of chondrogenesis during cartilage differentiation^155^. Similarly, GPC1 and 3 were able to inhibit BMP signaling and the osteogenesis mediated by BMP2 in human primary cranial suture mesenchymal cells^22^. BMP2 is also known to be regulated by perlecan. In contrast, perlecan has been shown in vitro to stimulate chondrogenic differentiation by modulating BMP2^156^ and to improve osteogenesis by increasing BMP2 signaling^157^. Finally, agrin has been demonstrated to play a role in osteoblast differentiation by regulating BMP signaling pathways^158^.

#### Implication of HSPGs in FGF signaling pathways

The human FGF family is composed of eighteen secreted proteins that can induce various different actions by binding one of the four FGF receptors (FGFR)^159^. The FGF signaling is known to regulate stem cell pluripotency and differentiation^160,161^. Numerous studies have demonstrated the implication of FGF in embryogenesis^105,162^ and in development/regeneration of various organs^161^ such as bones^163,164^, spinal cord^165^ or lung^166,167^.

The FGF signaling pathways need a fine regulation for a correct morphogenesis or regeneration of organs and numerous studies have proven the role of HSPGs in these processes.

#### Regulation of FGF signaling by HSPGs modulates embryonic stem cell fate

Studies performed on mouse embryonic stem cells have highlighted the importance of the HS chain sulfation during the formation of the mesoderm^152^. Indeed, the HS chain sulfation has been demonstrated to regulate FGF signaling involved in the mouse embryonic stem cell differentiation^110^. Johnson and collaborators have shown in culture of mouse embryonic stem cells that HS sulfation increases the FGF2 cell surface binding, inducing a cell differentiation into neural progenitor cells^168^. In the case of zebrafish development, GPC4 was demonstrated to induce the migration of lateral line collective cells during embryogenesis by the regulation of FGF signaling^109^ and agrin is necessary for the retina formation of zebrafish probably by the regulation of FGF8 signaling^169^.

Other studies, carried out on neuroepithelial cells have proven evidence of the role of different HSPGs on FGF signaling regulation during embryogenesis. In the case of neural stem cell proliferation, survival and differentiation, it has been shown that the HS chains of HSPGs and their sulfation are essential for the regulation of FGF distribution and binding and thus for the correct neuroepithelial tissue development^170–174^. The regulation of FGF signaling by GPCs seem to be highly implicated in the brain development processes. For example, GPC1 was shown to regulate the interaction between FGF17 and its receptor during early neurogenesis controlling the size of the mouse brain^175^. In mouse neuroepithelial cells, the HS chains of GPC4 are able to sequestrate FGF2 to prevent the binding with its receptor leading to the maintenance of their proliferative stem cell phenotype^176^. In contrast, in the case of Xenopus neurulation, GPC4 was demonstrated to bind FGF2 to facilitate the binding with its receptor regulating the dorsoventral forebrain patterning^177^. As last example for GPCs, a study conducted on mouse cerebral cortical development suggests a role of GPC6 in the regulation of FGF2 signaling during this process^178^. Perlecan was also shown to be important in the regulation of the brain development modulated by FGF signaling pathways. In the case of Drosophila, Trol is able to mediate FGF signaling to activate neural stem cell division^134^. In the case of vertebrate, FGF2 signaling is modulated by perlecan to regulate proliferation and differentiation of neural stem cells^135,179,180^. Agrin was demonstrated to regulate GABAergic and glutamatergic neuron development in zebrafish forebrain by the modulation of FGF signaling^137^.

#### Regulation of FGF signaling by HSPGs modulates vertebrate adult stem cell fate

Numerous studies have provided evidence on the role of HSPGs in vertebrate adult stem cell differentiation modulated by FGF signaling pathways. SDC3 was shown to induce the proliferation of chick chondrogenic progenitors by modulation of FGF2 signaling^181,182^. The regulation of FGF2 signaling by the HS chains of HSPGs was demonstrated to be essential for the regulation of mouse muscle satellite cells and myoblasts differentiation^183,184^. SDC3 is able to facilitate the interaction between FGF2 and its receptor leading to the repression of myogenic differentiation of murine skeletal myoblasts^185^. In contrast, GPC1 was reported to sequester FGF2, preventing its binding to its receptor, to promote mouse muscle differentiation^186^. The last following example highlights the role of HSPGs on skin progenitor cell fate modulated by FGF signaling. GPC1 is expressed by the epidermis keratinocytes mainly in the basal layer where the progenitors are present. In parallel, GPC1 cleavage is able to decrease human keratinocyte proliferation induced by FGF2^187^. These results tend to indicate a role of GPC1 in skin precursor cell proliferation during skin regeneration.

#### Implication of HSPGs in modulation of other signaling pathways

Additional roles of HSPGs in the regulation of vertebrate embryonic stem cell fate are described (Table 2) in particular during the central nervous system development^188–192^. There is also evidence that HSPGs play a critical role in the differentiation of hematopoietic progenitors and stem cells^193–199^.

In case of adult stem cell fate, the role of HSPGs on chondrogenesis and cartilage formation was confirmed by other studies conducted on HS chains^200^ and perlecan^201^. Moreover, the HS chains have been shown to potentiate the TGFβ3 signaling promoting chondrogenic differentiation of human mesenchymal stem cells^202^.

The sulfation of HS chains is crucial for a correct mouse colonic epithelial cell differentiation^203^. SDC3 interacts with Notch and regulates mouse adult myogenesis^204^.

As last examples, the role of HSPGs on the regulation of skin progenitor cell differentiation are presented. The first evidence was proven by the HSPG distribution within the epidermis which depends on the state of keratinocyte differentiation^205,206^. For example, SDC1 is weakly detected in the basal layer of epidermis and highly expressed in the suprabasal layers^207^. On the contrary, GPC1 is distributed throughout the epidermis but preferentially in the basal layer^187^. Few studies have been conducted on the pathways regulated by HSPGs involved in epidermis progenitor cell differentiation (see FGF subsection). Another example is the regulation of adhesion and early differentiation of keratinocytes progenitor cells by the formation of a complex between transient receptor potential canonical channel 4 (TRPC4) and SDC, demonstrating in Caenorhabditis elegans^208^.

All these examples demonstrate the role of HSPGs on the stem cell fate *via* the regulation of growth factor distribution, sequestration and downstream signaling pathway. Moreover, they highlight the opposing effects that HSPGs can exhibit on stem cell behaviors, either by favoring the maintenance of pluripotency or by promoting differentiation. Finally, these examples also show the different mechanisms of action of HSPGs. Indeed, they can sequester growth factors to prevent or facilitate the interaction with the receptors thanks to their HS chains or they can promote the interaction by their core protein.

### Importance of HSPG in hair follicle stem cells differentiation

#### Major signaling pathways involving in hair follicle stem cell differentiation

**Fig. 5:**
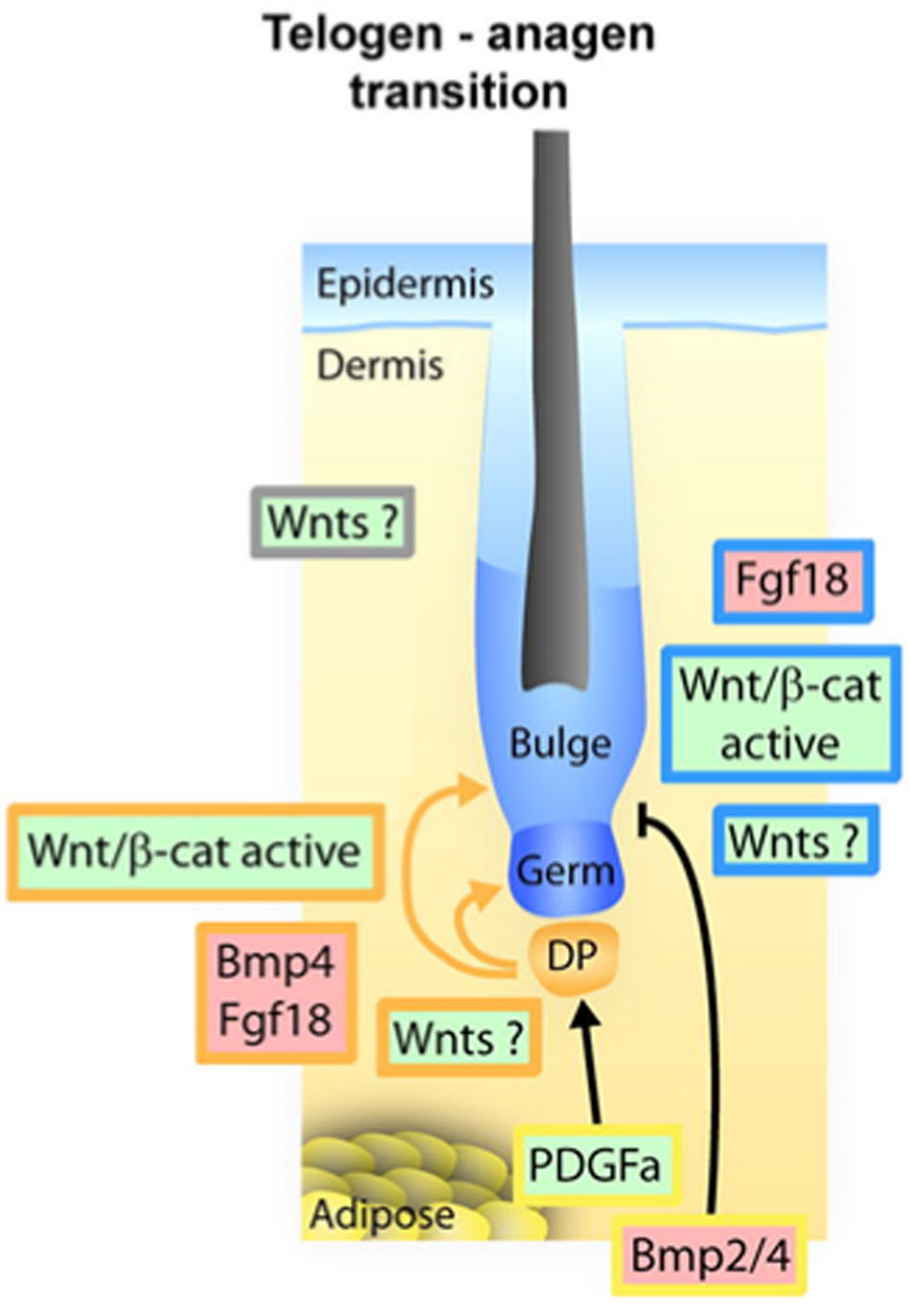
Regulation of stem cell differentiation during the telogen to anagen transition. The dermal papilla secretes Wnts which activate the stem cells of the bulge and the progenitor cells of the secondary germ. In contrast, the dermal papilla also produced BMPs promoting quiescence of these cells and prevent their activation. (This Figure was published in Sennett and Rendl, 2012^211^; Copyright Elsevier).

In the case of hair follicles, the major signaling pathways of growth factors (Wnt, Shh, BMP and FGF) are particularly important to regulate stem cell maintenance or differentiation during hair cycle and hair shaft growth (Fig. 5).

The platelet-derived growth factor (PDGF) secreted by fat cells activates the Wnt pathway within the DP^209^. In parallel, an inhibition of the BMP pathway is observed in the adipose macro-environment and in the dermal papilla^210,211^. The BMP pathway is active throughout the telogen phase and allows the maintenance of quiescence of SHG progenitor cells.

At the end of the telogen phase, various BMP inhibitors are secreted from the dermal papilla, particularly Noggin^211^ (Fig. 5). With the BMP pathway inhibition, secretion of Wnts from the dermal papilla has an activating effect on SHG progenitor cells and bulge stem cells at early anagen phase. Then, the Wnt/β-cat pathway will be activated in SHG progenitor cells and in bulge stem cells^211^.

The activation of these cell types leads to the secretion of different growth factors regulating the hair stem cell differentiation and the formation of a new hair shaft. For example, the production of insulin-like growth factor 1 (IGF1) by the DP controls the proliferation and the differentiation of SHG progenitor cells to regenerate the hair matrix^212^. The keratinocyte growth factor (KGF or FGF7), produced by the DP, stimulates the hair matrix cells, which then produce keratinocytes to form the new hair shaft^212^. The secretion of HGF by the DP promotes the elongation of the hair shaft^213^. Hedgehog (Hh), secreted by the hair matrix cells, stimulates the bulge stem cells to provide a new pool of SHG progenitor cells necessary for the next growth cycle^214^. In parallel, angiogenic pathways are also involved. The fibroblasts of the DP as well as the keratinocytes of the matrix and the ORS secrete VEGF which induces the formation of new blood vessels which provide the nutrient supply necessary for the formation of the hair shaft^212,215,216^.

Other growth factors are very important for the anagen-catagen transition. For example, epithelial growth factor (EGF) and FGF5 are necessary for this transition^217^. TGFβ and brain-derived neurotrophic factor (BDNF) inhibit HGF expression and VEGF secretion, respectively^14^. Moreover, the inhibition of Noggin and Wnt pathway signaling, as well as FGF18 and BMP2, 4 secretion by the dermal papilla, promote the quiescence of bulge stem cell^14,211^.

This brief summary provides an overview of the intense complexity of the crosstalk required for the maintenance and regulation of the hair cycle. This level of regulation is permitted by multiple growth factors, several signaling pathways and includes numerous different cell types. These regulations are still poorly understood but some studies provide evidence of the implication of HSPGs in these regulatory processes.

#### Involvement of HSPGs in hair follicle stem cell differentiation

In this section, the roles of HSPGs in hair follicle stem cell fate are presented (Table 2).

Studies have been conducted on the distribution of HSPGs in the different hair structures during hair growth cycle. During the anagen phase, HS chains are detected in the basement membrane, connective tissue sheath and the dermal papilla of hair follicle^17,218–221^. Experiments conducted on specific HSPGs have demonstrated that perlecan is expressed in basement membrane and the dermal papilla;^17^ SDC1 is expressed in the ORS, in the hair shaft and lower in the IRS and dermal papilla;^15,17^ GPC1 is expressed in the hair matrix (more strongly in the differentiation zone) and less in the hair shaft^16^.

Moreover, it has been shown that HSPG distribution in hair follicle evolves during hair growth cycle. For example, it has been demonstrated that perlecan is still expressed in basement membrane and connective tissue sheath during catagen phase but in dermal papilla a decrease of its expression is observed in late catagen^17^. Bayer-Garner and collaborators have shown that SDC1 expression in ORS decreases in telogen phase^15^. In contrast, the distribution of GPC1 in the hair matrix and hair shaft seems to be the same all along the hair cycle^16^. Moreover, it has been demonstrated that a fine regulation of HSPG sulfation is necessary for a correct formation of hair shaft^19^. It is interesting because the type and/or the degree of sulfation vary during hair cycle^16^. These studies suggest a role of HSPGs on hair follicle stem cell fate. Indeed, the tissular or cellular distribution of growth factors is associated with the expression of HSPGs during Drosophila embryogenesis^20,24,151^, bone formation^222^ or skin regeneration^187,208^ where HSPGs are demonstrated to regulate the pathways involved during these processes.

One another evidence is the fact that other proteoglycans, such as CSPG or DSPG regulate the signaling pathways involved in hair follicle stem cell fate. In particular, versican expressed in the dermal papilla is well described. Several studies showed the ability of versican to induce anagen phase and hair inductivity^219,223^ *via* the Wnt/β-cat pathway^224^. In addition, decorin was shown to be an anagen inducer probably by downregulating TGFβ signaling^225^. The similarity between the mechanisms of action of HSPGs and other sulfated proteoglycans emphasizes the role of HSPGs in the regulation of signaling pathways involved in hair follicle stem cell differentiation.

To conclude this section, the key role of HSPGs in the regulation of hair growth cycle and hair shaft formation is well established. Unfortunately, despite several studies carried out on the distribution of HSPGs on hair follicles, only few studies have investigated the mechanism by which HSPGs regulate these processes and which growth factor and signaling pathways are involved. Further works are necessary to better understand HSPG mechanisms of action and to develop HS proteoglycan-based therapies for hair disorders.

#### HSPGs as therapeutic targets for androgenetic alopecia

Androgenetic alopecia accounts for 90% of alopecia cases and affects 50% of women and 80% of men in their lifetime^226^. In men, it manifests as hair loss in localized areas^227^ and diffused loss in women^228^. According to Grand View Research, Inc., the global alopecia market size was valued in 2020 at USD 7.6 billion. From 2021 to 2028, it is expected to expand at a Compound Annual Growth Rate of 8.1% and to reach USD 14.2 billion. Androgenetic alopecia is due to an excessive supply of androgen to the dermal papilla causing physiological disruptions in hair follicles^229^. As described in the previous chapter, the Wnt signaling is crucial for hair stem cell differentiation and for the growth of the new hair shaft. In vitro, androgens were shown to inhibit the production of Wnt by dermal papilla cells^230^. This type of inhibition could explain the deregulation in vivo of the various growth factors secreted during the anagen phase. These deregulations during the anagen phase promote hair follicle miniaturization^231^. For example, the inhibition of IGF1 and KGF promotes hair thinning^231^. Shh inhibition disrupts the SHG formation and VEGF inhibition disrupts perifollicular revascularization. In addition, it has been shown that androgens stimulate the secretion of TGFβ^231^ and IL-6^232^ that induces premature transition to the catagen phase. In other context, androgens modulate the HSPG expression. For instance, the SDC1 expression decreased in the mouse mammary tumor cells after incubation with testosterone^233^. In addition, the steroid hormone estradiol has been demonstrated to modulate SDC3 expression and distribution playing a role in rat uterine growth^234^.

Currently, two major drug treatments exist, Minoxidil (lotion) and Finasteride (oral tablet) as well as surgical and low-level laser treatments^227,235^. Several cosmetic active ingredients, such as Stemoxydine, have also been developed and packaged in the form of shampoo, hair lotion, etc. These active ingredients reduce inflammation and/or improve micro-vascularization to promote the growth phase of the hair shaft and stop hair loss (Table 3). These drug treatments act to restore cellular signaling pathways dysregulated in androgenetic alopecia. For example, Minoxidil was demonstrated to activate the Wnt pathway in the mouse dermal papilla in vivo and in human dermal papilla cells in vitro^236^. This activation may be related to a stimulation of adipocyte precursors to secrete PDGF that activates the dermal papilla^237^. The stimulation of Wnt signaling in dermal papilla is able to restore the secretion of IGF1, HGF, and VEGF^238^. Furthermore, during the catagen phase, Minoxidil was shown to inhibit TGFβ-induced apoptosis in matrix TA cells and to increase the Bcl-2/Bax ratio protecting cells from apoptosis^239^.

**Table 3:** Mode of administration, mode of action and side effects of alopecia treatments and active ingredients.

Treatments/ Cosmetic active ingredients	Application/Administration	Mode of action	Side effects	References
Minoxidil	Topical application	Increases hair growth by prolonging anagen duration. Other possible mechanisms of action: stimulation of angiogenesis throught VEGF	Inflammatory skin reaction, eczema and allergies	York et al. 2020
Finasteride	Oral administration	Prevents androgen dependent miniaturization of hair follicles by competitively inhibiting 5-alpha-reductase	Sexual disorders	York et al. 2020
Stemoxydine	Topical application	Mimics the effects of the hypoxic environment essential for hair stem cells	No observable side effects	https://www.anabolichealth.com/stemoxydine-review/
Nourkrin	Oral administration	Intake of proteoglycan to the hair follicle leading to re-establishment of the hair follicle metabolism	No observable side effects	Thom^243^

Some studies have highlighted the link between hair growth disorder and the alteration of the expression and/or the distribution of proteoglycans. In particular, it has been shown that the dermal papilla of hair follicle isolated from a bald area presents a lower gene and protein expression of versican compared to those isolated from a hairy area^240^. The alteration of the expression and/or the distribution of HS proteoglycans in case of hair loss could be explained by the fact that androgens are able to modify the HSPG expression. Finally, evidence demonstrates the role of HSPGs in hair follicle stem cell fate during hair growth cycle. Altogether, these data highlight the role of HSPGs is the hair follicle physiopathology and make HSPGs as interesting targets for treatment of androgenetic alopecia. Despite this fact, few studies have been conducted to develop proteoglycan-based treatments for hair loss (and no one on HSPG-based treatments). After many years of proteoglycans related studies on their potential therapeutic development, Wadstein and Thom were the first to demonstrate the effect of proteoglycan concentration or synthesis in hair follicle growth^18^. Based on a clinical study, a cocktail of proteoglycans (oral administration) was shown to induce changes in hair follicle structure^241^. A specific cocktail of proteoglycans (Nourkrin), rich in lectican and decorin, was formulated. Clinical studies, conducted on patient with hair loss, have proven its efficacy on hair growth in monotherapy^242,243^ or in add-on treatment^244^ by increasing the hair count and reducing hair loss. Nourkrin is developed as oral treatment. It seems that the oral ingestion of proteoglycans leads to an increase of proteoglycan concentration in the hair follicles by direct deposition and/or synthesis of proteoglycans without secondary effect^18^. Nourkrin is a drug-free bioactive proteoglycan formula, based on natural ingredients. Studies are required to develop topical HS proteoglycan formula. However, some studies highlight possible vehicle or formula to induce the HS proteoglycan skin penetration such as polymersomes, vesicular nanocarriers, liposomes, nanoparticles, topical solutions and gels^245^. For example, in the case of wound healing, proteoglycans were topically applied using 30% glycerol formula^246^. Minoxidil was shown to present long term benefits although it has been demonstrated to induce a transient post-treatment hair shedding^247^. Interestingly, Nourkrin treatment was shown to do not induce post-treatment hair shedding with an uncharacterized molecular mechanism^18^. Nevertheless, it has been demonstrated that proteoglycans purified from salmon cartilage promote the wound healing of dermal fibroblasts^248^ and induce their proliferation, by the MAPK/ERK signaling pathway activation^249^. Similarly, a versican treatment on fibroblast stimulates their proliferation^250^. Interestingly, the application of proteoglycans purified from salmon cartilage on hematopoietic progenitor cells induce their differentiation into progenitor cells for granulocyte-macrophages, erythrocytes and/or megakaryocytes^251^. All these studies provide evidence that application or oral administration of proteoglycans can modulate the cell fate by the regulation of signaling pathways. Moreover, the first studies conducted on proteoglycan-based therapy for hair loss are promising. The mechanism of action of HSPGs on signaling pathway involved in hair follicle stem cell fate remains to be elucidated in order to develop HSPG-based treatment for hair loss.

## Conclusion

It is clear that our knowledge on the role of HSPGs on stem cell fate becomes greater by the day. Several publications and reviews have reported the capacity of HSPGs to modulate signaling pathways in stem cells in various different ways. Indeed, they can interact directly with the growth factors and/or their associated receptors by their core protein or their HS GAG chains. This interaction can facilitate the binding between receptor and growth factor or can inhibit the pathway by the sequestration of the growth factor. Moreover, membrane HSPGs can be cleaved to favor long-range activity of a growth factor. The understanding of the molecular mechanisms of action of HSPGs on stem cell fate highlights their key role on the regulation of organ morphogenesis, homeostasis and regeneration. In the case of hair follicle stem cells, there is still a long way to characterize the role of HSPGs. Indeed, no studies have reported a link between HSPGs and growth factors/signaling pathways involved in hair follicle stem cell fate. Moreover, few studies have shown a regulation of hair follicle stem cells by other proteoglycans (versican, decorin). Evidence on the role of HSPGs on the regulation of signaling pathways involved in hair follicles stem cell differentiation are proven by several studies that have demonstrated changes in the distribution of HSPGs during hair growth cycle and by the fact that HSPG sulfation is necessary for hair shaft growth. Currently, the Food and Drug Administration (FDA) and the European Medicines Agency (EMA) have only approved the topical Minoxidil and the oral Finasteride as treatment of androgenetic alopecia. These two drugs have demonstrated their ability to induce hair regrowth. However, both present side effects limiting their efficacy in a still unclear mechanism. In particular, the Finasteride is known to induce sexual disorder when the oral use is prolonged^245^. The Minoxidil topical application required specific formulation known to induce inflammatory skin reaction, such as eczema and allergy in case of repetitive application^247^. The fact that the treatment of alopecia requires long periods of application or oral administration represents the greatest limitation of these treatments. The Nourkrin oral tablet administration has shown good results on hair growth. Drug-free, it does not induce any side effect even during long period of application^243^. The intake of proteoglycan to the hair follicle leads to re-establishment of the hair follicle metabolism. The studies of the role HS proteoglycans in the regulation of the hair follicle metabolism will contribute to mastering the mode of action of the treatment based on HSPG delivery or to develop therapies targeting HSPGs. Proteoglycan-based treatment by oral delivery of proteoglycans appears to be a promising method to tackle androgenetic alopecia. Indeed, it has been shown to increase the hair counts on scalp. This kind of treatment can be applied with HSPGs oral delivery or topical application on scalp. Another way of investigation would be a treatment able to regulate the cellular expression of HSPGs to modulate their local distribution in hair follicles.
